# An 11-Year-Old Child with Autosomal Dominant Polycystic Kidney Disease Who Presented with Nephrolithiasis

**DOI:** 10.1155/2012/428749

**Published:** 2012-04-11

**Authors:** Fatih Firinci, Alper Soylu, Belde Kasap Demir, Mehmet Turkmen, Salih Kavukcu

**Affiliations:** ^1^Department of Pediatrics, Dokuz Eylul University, 35340 Izmir, Turkey; ^2^Department of Pediatric Nephrology, Dokuz Eylul University, 35340 Izmir, Turkey; ^3^Department of Pediatrics, Gaziantep Education Hospital, 27560 Gaziantep, Turkey

## Abstract

Patients with autosomal dominant polycystic kidney disease become symptomatic and are diagnosed usually at adulthood. The rate of nephrolithiasis in these patients is 5–10 times the rate in the general population, and both anatomic and metabolic abnormalities play role in the formation of renal stones. However, nephrolithiasis is rare in childhood age group. In this paper, an 11-year-old child with autosomal dominant polycystic kidney disease presenting with nephrolithiasis is discussed.

## 1. Introduction

Autosomal dominant polycystic kidney disease (ADPKD) is the most common hereditary kidney disease that is responsible for 8 to 10% of end-stage renal disease in adults [1, 2]. Although the disease might be diagnosed in the newborn or early childhood periods, the symptomatic disease commonly presents during adulthood [1]. The prevalence of nephrolithiasis among ADPKD patients is 20% which is 5–10 times more than the general population [2]. In addition to anatomic factors, metabolic abnormalities have also been described in the pathogenesis of nephrolithiasis in these patients. Nephrolithiasis is rare during childhood and commonly develops after the age of 20 [3]. However, in patients with known ADPKD presenting with flank pain, nephrolithiasis must be taken into consideration [4]. In this paper we describe an 11-year-old child with ADPKD who was presented with bloody urine and flank pain. He was determined to have nephrolithiasis and hypocitraturia as a predisposing factor.

## 2. Case Report

**Figure 1:**
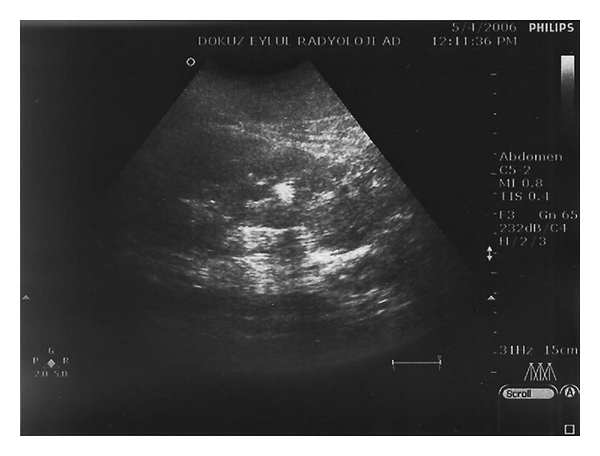
Cysts and calculi appear on USG.

An 11-year-old boy presented with the complaints of abdominal pain and bloody urine following a mild abdominal trauma. Physical examination was normal other than left costovertebral angle tenderness including anthropometric measurements and blood pressure. Urinalysis showed 3+ blood and 1+ protein with many isomorphic erythrocytes in the microscopic evaluation of the sediment. Serum biochemistry including renal and liver functions, electrolytes, calcium, phosphorus, and alkaline phosphatase was normal. Abdominal ultrasonography showed that both kidneys were larger than normal and had numerous millimetric cysts. There were bigger cysts at the upper pole of the right kidney and upper and lower poles of the left kidney reaching 10 mm, 12 mm, and 13 mm in diameter, respectively. In addition, there were numerous millimetric calculi in both kidneys with 3 large calculi in the right (8, 5, and 5 mm) and one in the left (9 mm) kidneys (Figure 1).

The patient's family history revealed that his father, paternal uncle, and aunt had polycystic kidney disease. Furthermore, his grandfather deceased due to chronic renal failure, and his uncle was undergoing chronic hemodialysis program. All affected family members had renal stone disease as well.

Metabolic investigations for urolithiasis were shown in Table 1. All test results were normal except urinary citrate excretion which was at the lower limit of the normal range.

The patient was hospitalized, and parenteral hydration was performed. His abdominal pain was controlled with paracetamol. Potassium citrate (2 mmol HCO_3_^−^/kg/day) was prescribed for prophylaxis of nephrolithiasis. He passed a stone in his urine at the 2nd month of followup. Stone analysis by X-ray diffraction method was consistent with calcium oxalate calculus. Extracorporeal shock wave lithotripsy was planned for the remaining stones.

## 3. Discussion

ADPKD is the most common hereditary kidney disease that is responsible for 5 to 10% of end-stage renal disease in adults. It has been shown that PKD1 (chromosome 16p13.3) and PKD2 mutations (chromosome 4q13q23) are causative in 85% and 15% of patients, respectively [1, 2]. In our case, mutation analysis was not performed. However, it has been reported that determination of 2 cysts in a single kidney or one cyst in each kidney in a patient under 30 years of age in a known ADPKD family is sufficient for diagnosis [5]. Thus, our patient was considered to have ADPKD.

In ADPKD, along with the cystic alterations in the kidneys, various extrarenal findings such as cysts in the liver, spleen and ovary, mitral valve prolapses, intracranial aneurysms, diverticulosis in the colon, and abdominal hernias may be seen [4, 6]. Abdominal ultrasonography did not reveal extrarenal cysts in our patient. On the other hand, we did not screen for the presence of intracranial aneurysms as the family history did not reveal any cerebrovascular event [7].

Abdominal pain is a common symptom in ADKPD. Acute pain may result from bleeding into the cysts, obstruction due to calculi,s or infection. Chronic abdominal pain is associated with the pressure caused by the kidneys and/or cysts. Nonnephrotoxic analgesics or cyst decompression or sclerosis may be applied in the treatment of chronic pain [1]. In our case, abdominal pain and hematuria were probably related to the presence of calculi. Hematuria disappeared spontaneously, while the pain responded to paracetamol.

The prevalence of urolithiasis in ADPKD is around 20–36 percent. Approximately half of the calculi is calcium oxalate and the other half consists of uric acid [3]. However, urolithiasis is rare in childhood and is usually seen after the second decade of life. In a study of 82 ADPKD patients, nephrolithiasis was detected in 23 (28%) patients. Forty percent and 60% of the cases were 20 to 40 years and over 40 years of age, respectively. None of the patients below 20 years of age had urolithiasis [8]. Our case is interesting in that he had 3 large and many small calculi while he was just at 11 years of age.

Increased risk of urolithiasis in ADPKD is associated with both intrarenal anatomic obstruction and urinary tract infections. On the other hand, metabolic abnormalities frequently accompany urolithiasis. Most common metabolic abnormality is hypocitraturia, while hypercalciuria and hyperuricosuria are having also been reported in some cases [3]. Ultrasonographic and metabolic parameters were compared in ADPKD patients having and not having renal stone disease. It was reported that patients having calculus had more and bigger cysts, while they had lower glomerular filtration rate, urine volume, urine phosphate, magnesium, citrate, and potassium levels. In both groups of patients, the rate of hypocitraturia was approximately 60 percent [9]. Our patient is probably prone to the development of urolithiasis due to the presence of numerous renal cysts and hypocitraturia. On the other hand, it has been reported that there are phenotypic differences in ADPKD families. The prevalence of hypertension and hernia along with patient and renal survivals differs between the families [10]. All effected family members of our patient have renal stone disease. This implicates that there may be a hereditary predisposition for renal calculus disease in this family.

In conclusion, in patients with ADPKD, multiple factors contribute to the development of urolithiasis including anatomic, metabolic, and hereditary abnormalities. Presence of nephrolithiasis in this 11-year-old child indicates the importance of the evaluation and followup of even young children with ADPKD for renal stone disease.

## Figures and Tables

**Table 1:** Metabolic evaluation for nephrolithiasis.

Test	Result
24-hour urine	
(i) Oxalate	(i) 0.28 mmol/1.73 m^2^/24 h (*N*: < 0.56)
(ii) Calcium	(ii) 3.2 mg/kg/24 h (*N*:< 4)
(iii) Uric acid	(iii) 9.4 mg/kg/24 h (*N*: < 10)
(iv) Citrate	(iv) 2.0 mg/kg/24 h (*N*: > 2)
(v) Cystine	(v) 35 mg/1.73 m^2^/24 h (*N*: < 60)

Serum	
(i) HCO_3_	(i) 24 mmol/L (*N*: 22–28)
(ii) Uric acid	(ii) 4.2 mg/dL (*N*: 1.7–5.8)
(iii) Calcium	(iii) 10.1 mg/dL (*N*: 8.8–10.8)
